# EPPRD: An Efficient Privacy-Preserving Power Requirement and Distribution Aggregation Scheme for a Smart Grid

**DOI:** 10.3390/s17081814

**Published:** 2017-08-07

**Authors:** Lei Zhang, Jing Zhang

**Affiliations:** College of Computer Science and Technology, Harbin Engineering University, Harbin 150001, China; zhangjing@hrbeu.edu.cn

**Keywords:** smart grid, smart meter, privacy-preserving, power requirement and distribution, homomorphic aggregation, hash message authentication code

## Abstract

A Smart Grid (SG) facilitates bidirectional demand-response communication between individual users and power providers with high computation and communication performance but also brings about the risk of leaking users’ private information. Therefore, improving the individual power requirement and distribution efficiency to ensure communication reliability while preserving user privacy is a new challenge for SG. Based on this issue, we propose an efficient and privacy-preserving power requirement and distribution aggregation scheme (EPPRD) based on a hierarchical communication architecture. In the proposed scheme, an efficient encryption and authentication mechanism is proposed for better fit to each individual demand-response situation. Through extensive analysis and experiment, we demonstrate how the EPPRD resists various security threats and preserves user privacy while satisfying the individual requirement in a semi-honest model; it involves less communication overhead and computation time than the existing competing schemes.

## 1. Introduction

With the advance of information and communication, the transition of the traditional electrical grid into modern power system promotes the generation of Smart Grid (SG). The utility provider has also switched from the original arbitrary power distribution into the current bidirectional information exchange according to the user’s individual requirement and current power level. The bidirectional communication between the power companies and end users in SG facilitates mutual information exchange, by which SG has become a platform playing an important role in power generation, transmission, distribution, managing and monitoring system [1,2,3].

As one key element of SG, the advanced metering infrastructure (AMI) further develops the smart metering, smart billing, demand response system, fault-tolerance and attack monitoring, but also bring the risk of revealing user privacy [4,5]. To this end, privacy-preserving aggregation protocols has emerged in secure metering of SG since the aggregate sum for smart metering is computed without leaking user measurements. The existing scheme, such as in [6], presents a comparison of four concrete protocols for secure aggregation smart metering, namely interactive protocols, the Diffie–Hellman Key-exchange based protocol, Diffie–Hellman and Binary mapping based protocol, and low-overhead protocol. The last three protocols adopt secure and a relaxed Diffie–Hellman key exchange protocol that lowers the computation and communication overheads. However, this work does not consider smart meter authentication, while our scheme extends Diffie-Hellman-based authentication. Bartol et al. in [7] provide a privacy aggregation scheme in which messages of smart meters are concatenated to generate a single aggregated message. This scheme can preserve the privacy of every user. However, the aggregator must sequentially decrypt all messages in an aggregated message to generate the data total, which generates a lot of computational overheads. In order to reduce computational overheads after aggregation and prevent the middle nodes or aggregator from compromising the individual data, additional homomorphic encryption occurs, in which the encrypted individual measurements are added to generate the ciphertext of sum, and then is decrypted into the plaintext of the sum. In [8,9,10,11,12,13,14,15], the proposed homomorphic encryption does not need sequential decryptions at the aggregator, so aggregation time is short and overhead is low. Garcia et al. in [11] provide a privacy-preserving aggregation scheme without compromising individual data with secret sharing and Paillier homomorphic encryption. The aggregator is responsible for receiving, computing, and distribution of N nodes, but the number of homomorphic encrytions per user is linear. Li et al. in [9] deploy a distributed incremental aggregation approach in hop by hop (HBH) networks, which aggregates the node data of its children and relays them to its parents’ nodes. The scheme constructed a breadth-first traversal tree corresponding to the graph of the networks within a neighborhood. Erkin et al. in [15] realizes improved homomorphic aggregation scheme in which any user can aggregate total power consumption for all users at a time stage or a user smart metering for a series of time, and random numbers are added into every individual user to encrypt individual measurements. However, a lot of interaction among smart meters aggravates the whole computation burden.

In addition to Paillier homomorphic encryption, Diffie-Hellman-based, ElGamal and the BGN homomorphic encryption scheme are also used in homomorphic aggregation of SG data. [16] ensures the aggregator oblivious with the shared keys and uses Diffie-Hellman homomorphic encryption and distributed differential privacy. However, the aggregation sum must be achieved through the brute-force search; consequently, the decryption time is the square root of the length of the plaintext even when Pollard’s method is used. The blinding shares of zero to each user as private key involves a presentation for every user, and distributing new share keys when new nodes are added or existing nodes leave, which aggravates the computational burden on the system. The employed method in [5] encrypted user data homomorphically on demand using ElGamal encryption and transmited the data to the utility provider based on an HBH network communication model.

However, most of the above schemes do not provide individual user services; they only provide the total required power consumption and requirement to the utility provider. Therefore, the current research issue in SG is to study schemes that can meet individual demands and adjust the power distribution according to the current power level [17]. Lu et al. in [12] applied a multi-dimensional individual data aggregation method in an ETE (end-to-end) network model and reported that batch verification saves a considerable amount of communication overhead. User privacy was ensured in [17] by separating the users’ real identities from their fine-grained metering data; thus, attackers can discover either the user’s identity or their fine-grained metering information, but not both. However, [17] focused on the anonymity and privacy scheme and did not address authentication issues.

Existing authentication schemes [18,19,20,21,22] all include encrypted hash functions, especially hash message authentication codes (HMAC), which are applied to protect the integrity of messages against deliberate alterations. A simple authentication scheme was designed in [18] for upward communication that used digital signatures for downlink communications. The approach in [20] ensured secure bidirectional communications between the smart meters and the aggregator using bitwise exclusive-OR operations for encryption and a Lagrange interpolation formula for authentication.

Different smart grid applications have different network requirement in terms of data payloads, sampling rates, latency, and reliability [3]. As revealed in [3], in a smart grid environment, a communication network can be represented by a hierarchical multi-layer architecture, which is divided into several area networks (i.e., Home Area Network (HAN), Building Area Network (BAN), Industrial Area Network (IAN), Neighborhood Area Network (NAN), Field Area Network (FAN), and Wide Area Network (WAN)). A comprehensive and hierarchical structure for smart grid communications was proposed in [19,22] that used a three-layer network: HANs at the user level, BANs at the building level, and NANs at the substation level. In this configuration, different gateways are responsible for aggregating data, namely, the HAN Gateway (GW), BAN Gateway (GW), and NAN Gateway (GW), which reside in each corresponding layer of the network. Based on this hierarchical architecture, they proposed an authentication scheme based on computational Diffie–Hellman encryption to maintain data integrity. We adopt a lightweight authentication method in combination with our hierarchical network architecture to satisfy the scalability and the real-time and efficient communication requirement while preserving privacy.

We propose an efficient privacy-preserving power requirement and distribution aggregation scheme for a Smart Grid (EPPRD). The scheme focuses on securing the communications required to implement individual power requirements and distribution suited to the current power level, in which a lightweight, scalable authentication protocol is proposed for bidirectional communication based on hierarchical communication networks. The main contributions of this paper are as follows:

- It may be necessary to adjust a user’s power distribution in the next time slot to flatten demand peaks based on the power consumption in the current time slot, because power changes dynamically over time. During peak demand, the Control Center (CC) reduces the total distribution to users to adjust power consumption from peak time to non-peak time in the next time slot. Therefore, our demand message is divided into two parts: an individual user requirement based on RSA encryption for the next time slot and the total user consumption based on Paillier encryption in the current time slot, which is one significant reference of power distribution at the next time slot for the CC.
- To reduce the volume of transmitted traffic, we locate a regional concentrator in the BAN for regional storage, aggregation, transmission, and distribution. After the BAN receives the distributed regional power ratio from the CC, it immediately distributes individual power to the users according to the stored requirement and the distributed regional power ratio.
- To ensure message confidentiality and integrity, we employ the Public–Private, Paillier homomorphic cryptography and Hash-based Message Authentication Code authentication in the HAN Smart Meter (HSM), BAN Gateway (BGW), and NAN Gateway (NGW). This scheme can resist various attacks, such as replay attacks, man-in-the-middle attacks, eavesdropping attacks, and so forth. This scheme offers stringent security and reliability guarantees.
- The remainder of the paper is organized as follows. In Section 2, we introduce related work with EPPRD. In Section 3, we introduce an EPPRD communication model and security goal. In Section 4, we introduce the basic preliminaries such as Computational Diffie-Hellman (CDH) Problem and Paillier cryptosystem. In Section 5 we propose the EPPRD scheme and security analysis and proof. After that we present our performance analysis and discussion in Section 6 and Section 7, respectively. Finally, we draw conclusions in Section 8.

## 2. Related Work

Although multiple studies in [8,19,21,22,23,24,25,26,27] have already proposed methods to securely aggregate user measurements in SG, they have focused primarily on total user power aggregation rather than on individual information exchange between a user and a power utility, and such total aggregation is not suitable for modern individual demand-response characteristics. Some of the proposed methods are also vulnerable to various attacks because they lack a rigorous authentication process, and some are inefficient due to their high communication overhead.

In [19,22], the authors introduce a simple authentication scheme. Two parties (in HAN and BAN or NAN) establish a shared key using the Diffie-Hellman Technique, after the initial authentication, they generate HMAC signatures for all subsequent communications. However, these studies did not address the issue of privacy at all. A hierarchical communications architecture was also adopted in [21], which proposed an individual security billing scheme based on the hierarchical communications architecture. The user submits an encrypted power requirement to the aggregator. When billing, the user can show the CC the pre-submitted requirement and receives a reward or penalty. Although the scheme adopts a method similar to ours regarding the hierarchical communications architecture, HMAC authentication, and bi-directional communication, there are some differences between [21] and our study:

- Our scheme focuses on preserving the privacy of individual power requirement and distribution instead of on individual power billing. We adopt two different encryption modes for individual power requirement and distribution, while [21] employs only Paillier homomorphic encryption for its power requirement.
- Zhong et al. in [21] employ commitments to store an individual power requirement and transmits it upward through nodes to the CC, which generates excessive communication overhead, while we employ a regional concentrator to store and distribute the individual power requirement.
- From a security and data integrity perspective, [21] employs only one authentication key throughout the entire authentication process; however, as is well known, a user’s smart meter is more vulnerable to attack than a gateway is. Therefore, if the authentication key is compromised, all the subsequent authentication processes are vulnerable to a man-in-the-middle attack. Our scheme strengthens this aspect by adopting a stringent method of authentication between the HSM and the BAN Concentrator (BC) to reduce the vulnerability of the HSM. In our scheme, a new authentication key is generated randomly based on the Diffie-Hellman key establishment protocol in every communication session. In comparison with [21], we show that when the number of smart meters is very large, our protocol is more efficient and more stringent than competing schemes.

The study in [8] presents a secure privacy preserving aggregation method to protect the electricity consumption of an individual user. It can also resist internal attacks. However, it differs from ours in its encryption scheme, authentication, and trustable nodes. TTP is employed in [8], while we employ regional concentrators in the BAN layer.

Numerous authentication schemes have been proposed thus far [23,24,25]; however, all these schemes suffer from too many authentication steps, which cause high communication overhead and long delays. In this paper, these challenging issues are ameliorated [8] by proposing a robust, efficient, and lightweight message authentication scheme to ensure secure communications between the GWs. Our authentication scheme provides mutual authentication among smart meters located in different area networks in a hierarchical communication network. The proposed authentication scheme is based on the Diffie-Hellman key establishment protocol and keyed Hash-based Message Authentication Code (HMAC K) in [19].

Of course, there are quite a few studies that involve regional concentrators. Of these, [26,27] are closest to ours. The employed method in [26] provided a comprehensive performance analysis of the Split and Aggregated TCP (SA-TCP) scheme. It studies the impact of varying various parameters on the scheme, including the impacts of network link capacity and the buffering capacity of Regional Collectors (RCs), and it uses RCs as the SA-TCP aggregators. It is noted in [26] that RCs are trustable gateways that are installed at preselected locations in every region to route the meters’ data packets through a wide area network to the utility server. The study in [27] compares the performance of four different WSN architectures in terms of energy consumption, in which the CN (Concentrator Node) in the third presented architecture is similar to the regional concentrator in our scheme. A trustable regional concentrator should have some storage and processing capability to allow it to aggregate the periodically generated regional metering information and stores those values in its memory. Then, for example, at the end of the day, the concentrator could aggregate the information and send a summary message to the CC [27]. Using this approach, the traffic in the low-level network can be greatly reduced.

Based on this idea, we install also a trustable regional concentrator in the BAN layer, called a BAN Concentrator (BC). Each set of n meters establish n TCP connections with a BC, which is a gateway that acts as a regional aggregator and distributor. The difference from the two studies mentioned above is that the BCs in our scheme can not only store and sum up individual requirement but also aggregate individual consumption homomorphically to distribute individual power to the specific user.

## 3. Models and Goals

In this section, we formalize a system communication model, security goals, and attack model in EPPRD.

### 3.1. System Communication Model

**Figure 1:**
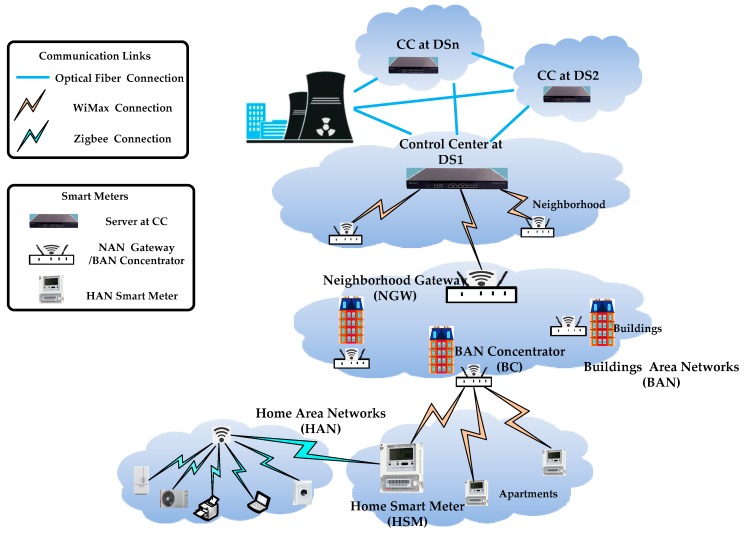
Hierarchical communication system model of smart grid.

The system communication model as shown in Figure 1 is based on the hierarchical communication architecture. In EPPRD, the communication network framework includes Neighborhood Area Network (NAN), Building Area Network (BAN), and Home Area Network (HAN). HAN, BAN, and NAN communicate through Wimax, and NAN connects the CC with optical fiber.

We use the HSM to represent HAN Gateway Smart Meter; the BC represents BAN Concentrator; NGW represents NAN Gateway; and the GWs stands for BC, and NGW below.

- CC: we assume CC is a highly trusted and powerful entity in charge of managing the whole system. Its duty is to initialize the system and to collect, process, analyze the real-time data, and provide power distribution according to the power level and real-time data.
- BC: we assume BC is a highly trusted gateway in charge of collecting, storing, aggregating, and distributing real-time data. BC can also store regional individual power requirements and aggregate regional power consumption and transmit it with regional requirement summation through the NAN to the CC and distribute individual power to every user according to the power ratio from the CC. BC needs enough secure storage, which can be used to handle the long-term keys described above and protect their private reading; this can be achieved, for example, by TPM chips to store the specific power requirements of HSM.
- NGW: NGW is a power gateway, which connects real-time data from BC and CC. The duty of NGW is to relay and aggregate real-time data. The duty of aggregation is aggregate the regional consumption data from BC, whereas the duty of relay is to relay the regional requirement data from BC in a secure way.
- HSM: we refer to HSM as a user with a smart meter and the HAN is made up of various smart applications. The real-time data of HSM is collected and processed by BC and transmitted into CC via NGW. Although HSM is tamper-resistant and interfering with measurements is not trivial, it is not as powerful as the gateway (e.g., BC, NGW), so it may be vulnerable to attackers.

For the sake of simplicity, we assume every set of m HSMs establish m TCP connections with a BC, every set of n BCs establishes n TCP connections with a NGW, and every set of p NGWs establishes p TCP connections with CC.

### 3.2. Security Goals

We have the following three security goals:

- Confidentiality. Authorized limitation to access data and encryption is critical to protect personal privacy and information—in other words, only the granted entity can receive the individual user data or access the databases of the GWs, i.e., an attacker cannot decrypt the communication flows between GWs and CC.
- Data integrity, authentication, and access control. Authentication and access control verify authorized communication entity and ensure access to the power information, which prevent an ungranted attacker from modifying and destructing the power data integrity and availability.
- Forward secrecy. Forward secrecy is a property of secure communication protocols in which compromise of long-term keys does not compromise past session keys.

To satisfy these secure goals, not only should every node be encrypted with cryptographic primitives but communication flows should be verified with an efficient and bidirectional authentication method.

### 3.3. Attack Model

We assume smart meters (e.g., HSM, NGW) are semi-honest (also known as “Honest but curious”) that faithfully follow all prescribed protocols and provide real measurements; however, they attempt to know as much data as possible. Although HSM is assumed to be tamper-resistant, we do not rule out the possible of data pollution (or DoS) attack. A data pollution attack is a kind of malicious participant attack where the attacker lies about their values, resulting in incorrect measurement results. It is not within the scope of this paper, but we would like to mention that one possible solution is interactive or non-interactive zero knowledgeproof.

- We consider the following possible attack types in EPPRD.
- External Attack: The external attacker tries to infer the individual information by eavesdropping on the communication and data flow from the HSM to the BC, from the BC to the NGW, and from the NGW to the CC.
- Internal Attack: Internal attackers are usually participants of the protocol (e.g., NGW) who may collude with as many compromised HSMs as possible to learn about the individual user’s privacy, or a curious HSM who attempts to infer the private data of another HSM.
- Man in the middle attack: The attacker forges or alters the communication data once he is authorized by any communication party, so the authentication key between HSM and BC should be different from that between BC and NGW to prevent the authenticated attacker from altering the communication data between BC and NGW.
- Replay Attack: Attacker tries to repeat or delay a valid data transmission while misleading the honest sender into thinking they have successfully finished the data transmission.

## 4. Preliminaries

In this section, we briefly provide some preliminaries for the security and authentication scheme used in EPPRD.

### 4.1. Computational Diffie–Hellman (CDH) Problem

The CDH problem is stated as follows: Given the elements $g^{a}$ and $g^{b}$, for unknown $a,b\in {Ζ}_{q}^{*},\;G=\langle g\rangle$ be a group of large prime order q, it is hard to compute $g^{\mathrm{ab}}\in G$. Based on the CDH assumption, the lightweight message authentication scheme is described in detail in [19] and is not repeated here.

### 4.2. Paillier Cryptosystem

The Paillier Cryptosystem was proposed in 1999 by Pascal Paillier and is one common homomorphic encryption that is widely used in privacy-preserving applications [28]. Concretely, the Paillier Cryptosystem is comprised of three algorithms: key generation, encryption, and decryption.

Key Generation: Given the security parameter κ, two large prime numbers p, q are first chosen, where |p|=|q|=κ. Make  N=pq, λ=lcm(p−1,q−1). Then define a function $L\left(u\right)=\frac{u-1}{N}$, after that choose $g\in {\mathbb{Z}}_{N^{2},\;}^{*}$ so make $\gcd\left(L\left(g^{\lambda }{\;\mathrm{mod}\;N}^{2}\right),\;N\right)=1$, Make $\alpha ={\left(L\left(g^{\lambda }{\;\mathrm{mod}\;N}^{2}\right)\right)}^{-1}\;\mathrm{mod}\;N.$ Then the public key is PK=(N,g), then the corresponding private key is SK=(λ,α).

Encryption: Given one message $m\in {\mathbb{Z}}_{N}$, a random $r\in {\mathbb{Z}}_{N,\;}^{*}$ the corresponding ciphertext can be calculated as $c=E\left(m,r\right)=g^{m}\cdot r^{N}{\;\mathrm{mod}\;N}^{2}$.

Decryption: Given one ciphertext $c\in {\mathbb{Z}}_{N^{2},\;}^{*}$ the corresponding message can be calculated as $m=D\left(c\right)=L\left(C^{\lambda }{\;\mathrm{mod}\;N}^{2}\right)\cdot \alpha \;\mathrm{mod}\;N$.

Homomorphic aggregation: For random parameter between the GWs and CC m, $m_{1},{\;m}_{2},{\;r}_{1},r_{2}$, then E($m_{1},r_{1}$)$\cdot E\left(m_{2},r_{2}\right)=E\left(m_{1}+m_{2},r_{1}\cdot r_{2}\right){\mathrm{mod}\;N}^{2},\;{\left(E\left(m_{1},r_{1}\right)\right)}^{m_{2}}$ = E ($m_{1}\cdot m_{2},{\;r}_{1}{}^{m_{2}}$) mod $N^{2}$.

Semantic Security: With the additional properties of the Paillier cryptosystem, the attacker cannot distinguish the ciphertext of plaintexts even if the plaintexts are the same. The semantic security is proved under the decisional composite residuosity assumption: Given N = pq, it is hard to decide whether an element in ${\mathbb{Z}}_{N^{2}}$ is an N-th power of an element in ${\mathbb{Z}}_{N^{2}}^{*}$ [18].

## 5. Our Scheme

### 5.1. System Initialization

For the given hierarchical communication system model in Figure 1, the CC can bootstrap the whole system. We randomly select one HSM node, one BC node, and one NGW node and denote them as ${\mathrm{HSM}}_{i}$, ${\mathrm{BC}}_{j}$, and ${\mathrm{NGW}}_{k}$, respectively. We assume that the ${\mathrm{BC}}_{j}$ has m HSM nodes, the ${\mathrm{NGW}}_{k}$ has n BC nodes, and the CC has p NGW nodes. The specific notations in our scheme are listed in Table 1.

The special initialization process is as follows:

- Given the security parameter  κ, CC first generates (p,q) by running Gen(κ), and calculates the Paillier Cryptosystem’s public key denoted, ${\mathrm{PK}}_{\mathrm{CC}}$ (n = pq, g) and the corresponding private key ${\mathrm{SK}}_{\mathrm{CC}}$ (λ,α), where p and q are two large prime numbers for which |p|=|q|=κ. The $<\mathrm{CC},{\;\mathrm{PK}}_{\mathrm{CC}}>$ is distributed to each node in the network model, and the ${\mathrm{SK}}_{\mathrm{CC}}$ is kept private;
- For each user’s smart meter, ${\mathrm{HSM}}_{i}$ generates a pair of public and private keys ${\mathrm{PK}}_{{\mathrm{HSM}}_{i}}$ and ${\mathrm{SK}}_{{\mathrm{HSM}}_{i}}$ respectively. Then, $<{\mathrm{HSMID}}_{i},\;{\mathrm{PK}}_{{\mathrm{HSM}}_{i}}>$ is stored at the control center and distributed to each user after initialization, while ${\mathrm{SK}}_{{\mathrm{HSM}}_{i}}$ is preloaded into the ${\mathrm{HSM}}_{i}$ and kept private.
- Each ${\mathrm{BC}}_{j}$ generates a pair of public and private keys, ${\mathrm{PK}}_{{\mathrm{BC}}_{j}}$ and ${\mathrm{SK}}_{{\mathrm{BC}}_{j}}$ respectively. Then, $<{\mathrm{BCID}}_{j},{\;\mathrm{PK}}_{\mathrm{BC}}{}_{j}>$ is stored at the control center and distributed to each user after initialization, while ${\mathrm{SK}}_{{\mathrm{BC}}_{j}}$ is preloaded into the ${\mathrm{BC}}_{j}$ and kept private.
- Each ${\mathrm{NGW}}_{k}$ generates a pair of public and private keys, ${\mathrm{PK}}_{{\mathrm{NGW}}_{k}}$ and ${\mathrm{SK}}_{{\mathrm{NGW}}_{k}}$, respectively. Then $<{\mathrm{NGWID}}_{k},{\;\mathrm{PK}}_{\mathrm{NGW}}{}_{k}>$ is stored at the control center and distributed to each NGW after initialization, while ${\mathrm{SK}}_{{\mathrm{NGW}}_{k}}$ is preloaded into the ${\mathrm{NGW}}_{k}$ and kept private.
- CC generates an authentication key, s, encrypts it with the BC’s and the NGW’s public ciphertext, and transmits it to the BC and NGW, respectively.

### 5.2. Upward Message Form

**Figure 2:**
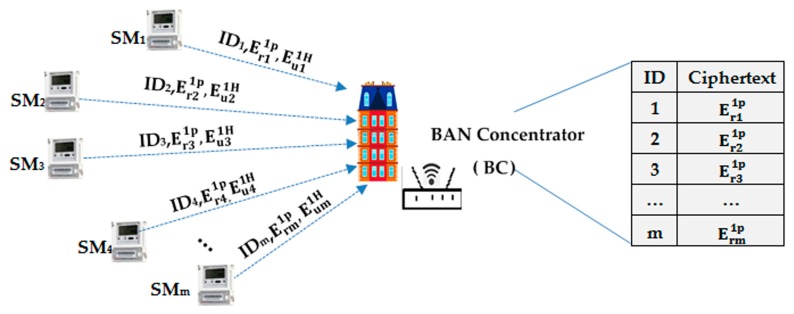
BC storage diagram.

In our scheme, the CC collects one power requirement and consumption instruction per collection period △, which include two parts: every user power requirement for the next time slot and the total power consumption for the last time slot. Respectively, these are the public RSA encryption part denoted as $E_{r}^{P}$, and the Paillier homomorphic encryption part denoted as $E_{u}^{H}$, as shown in Figure 2.

We encrypt each individual power requirement with public RSA encryption $E_{r_{i}}^{p}$ because the BC needs to store the encrypted individual requirement and decrypt it later to distribute power according to the power ratio at the power distribution phase.

In addition, individual power consumption requires summation to act as a reference for power distribution during the next time slot. For this, we employ homomorphic encryption $E_{u_{i}}^{H}$, which also prevents any intermediate nodes from leaking individual consumption.

${\mathrm{HSM}}_{i}$ computes the individual upwardly transmitted messages, ${\mathrm{msg}}_{i}$, as follows:

(1) $${\mathrm{msg}}_{i}=<{\mathrm{ID}}_{i},\mathrm{Len},E_{r_{i}}^{p},E_{u_{i}}^{H}>,$$

where $E_{r_{i}}^{P}$ represents the public encryption value of the requirement plaintext $r_{i}$ with ${\mathrm{PK}}_{{\mathrm{BC}}_{j}}$ and $E_{u_{i}}^{H}$ represents the homomorphic encryption value of the consumption plaintext $u_{i}$ with ${\mathrm{PK}}_{\mathrm{CC}}$.

The header includes two parts: ${\mathrm{ID}}_{i}$ denotes the sender ID and Len denotes the length of the public encryption part, which separates the non-homomorphic part from the homomorphic part.

As seen in Figure 2, we define every BC as both regional aggregator and distributor. They store encrypted individual power requirement, aggregate regional power consumption, and transmit it after regional requirement summation via NGW to the CC. They also distribute individual power to each user according to the power ratio from the CC.

### 5.3. Communication between $HSM_{i}$ and $BC_{j}$

#### 5.3.1. Authentication Part

**Figure 3:**
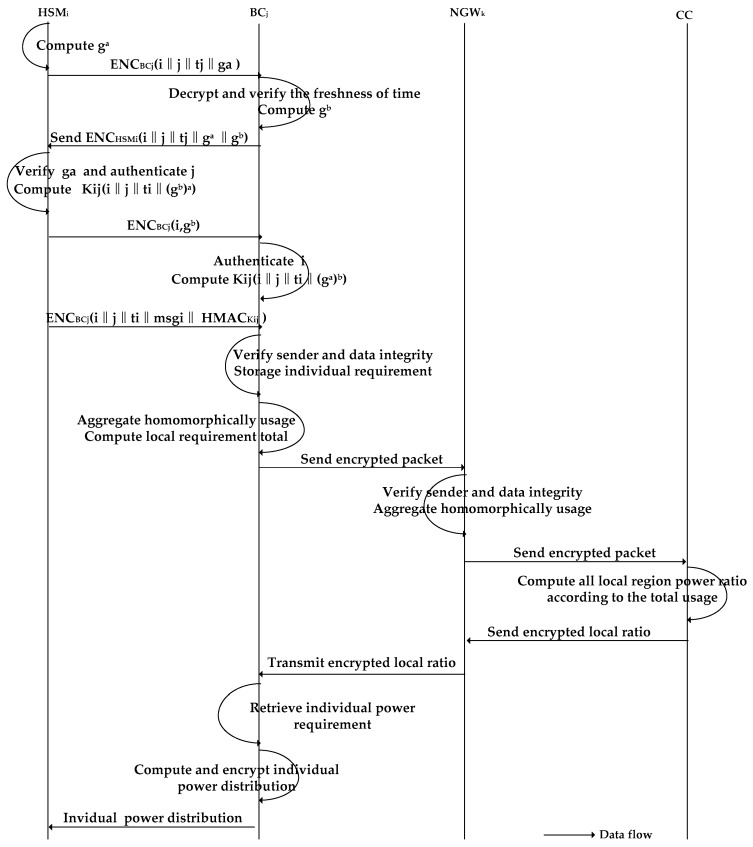
Authentication and data transmission process between GWs.

In the Related Work (Section 2), we mentioned that the authentication scheme in [21] is not sufficiently stringent because the only authentication key may be leaked. Therefore, we adopt an authentication protocol based on the Diffie-Hellman key-establishment protocol proposed in [19] between ${\mathrm{HSM}}_{i}$ and ${\mathrm{BC}}_{j}$. The specific processes are depicted in Figure 3.

- ${\mathrm{HSM}}_{i}$

${\mathrm{HSM}}_{i}$ selects a random number $a,b\in {\mathbb{Z}}_{q}^{*}$ from a positive integer in prime order. Let G=<g> be a group of prime numbers. Given $g^{a}$, ${\mathrm{HSM}}_{i}$ computes ${\mathrm{ENC}}_{{\mathrm{BC}}_{j}}\left(i∥j∥t_{i}∥g^{a}\right)$ (where $t_{i}$ is the current time slot) and transmits it to ${\mathrm{BC}}_{j}$.

- ${\mathrm{BC}}_{j}$

After receiving ${\mathrm{ENC}}_{{\mathrm{BC}}_{j}}\left(i∥j∥t_{i}∥g^{a}\right)$, ${\mathrm{BC}}_{j}$ first decrypts it with its private key, ${\mathrm{SK}}_{{\mathrm{BC}}_{j}}$, to verify the freshness of $t_{i}$. Then, it sends an encrypted response consisting of $g^{b}$ ${\mathrm{ENC}}_{{\mathrm{HSM}}_{i}}\left(i∥j∥t_{j}∥g^{a}∥{\;g}^{b}\right)$ to ${\mathrm{HSM}}_{i}$.

- ${\mathrm{HSM}}_{i}$

After receiving ${\mathrm{ENC}}_{{\mathrm{HSM}}_{i}}\left(i∥j∥t_{j}∥g^{a}∥{\;g}^{b}\right)$ from ${\mathrm{BC}}_{j}$, ${\mathrm{HSM}}_{i}$ first verifies the freshness of $t_{j}$. Then, it recovers $g^{a}$ and $g^{b}$ using its private key ${\mathrm{SK}}_{{\mathrm{HSM}}_{i}}$, If the recovered $g^{a}$ is correct, ${\mathrm{BC}}_{j}$ is authenticated by the ${\mathrm{HSM}}_{i}$. Then, with a and $g^{b}$, ${\mathrm{HSM}}_{i}$ can compute the shared session key $K_{\mathrm{ij}}=H\left(i∥j∥{\left(g^{b}\right)}^{a}\right)$, where $H:{\left\{0,1\right\}}^{*}\to {Ζ}_{q}^{*}$ is a secure cryptographic hash function, and computes the HMAC signature using $K_{\mathrm{ij}}$ as the key on i, j, $t_{i}$, and ${\mathrm{msg}}_{i}$ to form the Hash-based Message Authentication Code ${\mathrm{HMAC}}_{\mathrm{kij}}\left(i∥j∥t_{i}∥{\mathrm{msg}}_{i}\right)$. Finally, ${\mathrm{HSM}}_{i}\;$ sends ($g^{b},i)$ to ${\mathrm{BC}}_{j}$ to authenticate ${\mathrm{HSM}}_{i}$.

- ${\mathrm{BC}}_{j}$

After receiving $\left(g^{b},i\right)$, ${\mathrm{BC}}_{j}$ authenticates ${\mathrm{HSM}}_{i}$ and then computes $K_{\mathrm{ij}}=H\left(i∥j∥{\left(g^{a}\right)}^{b}\right)$ with the known $g^{a}$ and b.

#### 5.3.2. Upward Transmission

**Figure 4:**
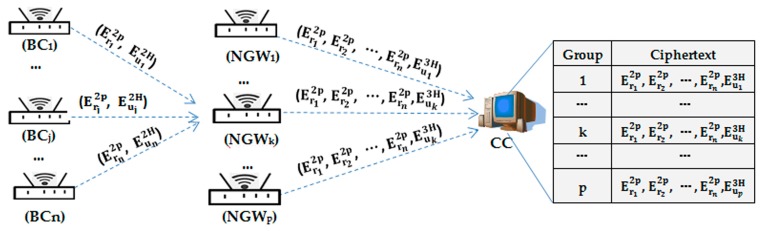
Message packet transmission process and storage.

After the authentication process between ${\mathrm{HSM}}_{i}$ and ${\mathrm{BSM}}_{j}$ is complete, the ${\mathrm{HSM}}_{i}$ transmits the message packet upward to ${\mathrm{BSM}}_{j}$. The specific transmission process is depicted in Figure 3 and Figure 4.

- ${\mathrm{HSM}}_{i}$

${\mathrm{HSM}}_{i}$ sends ${\mathrm{ENC}}_{{\mathrm{pk}}_{{\mathrm{BC}}_{j}}}\left(i∥j∥t_{i}∥{\mathrm{msg}}_{i}∥{\mathrm{HMAC}}_{\mathrm{kij}}\right)$ to ${\mathrm{BC}}_{j\;}$.

- ${\mathrm{BC}}_{j}$

${\mathrm{BC}}_{j}$ decrypts ${\mathrm{ENC}}_{{\mathrm{pk}}_{{\mathrm{BC}}_{j}}}$ $(i∥j∥t_{i}∥{\mathrm{msg}}_{i}∥{\mathrm{HMAC}}_{\mathrm{kij}}$) with ${\mathrm{SK}}_{{\mathrm{BC}}_{j}}$, verifies the freshness of $t_{i}$, and recomputes kij and ${\mathrm{HMAC}}_{\mathrm{kij}}$ based on i, j, $t_{i}$, and ${\mathrm{msg}}_{i}$ to verify the sender and the integrity of ${\mathrm{msg}}_{i}$. If it is not the same as the one attached, it requires the transmission to be resent.

After receiving all the messages from its child nodes, the ${\mathrm{BC}}_{j}$ aggregates all m $E_{u_{i}}^{1H}$ into $E_{u_{j}}^{2H}$ and decrypts all $E_{r_{i}}^{1p}$ with ${\mathrm{SK}}_{\mathrm{BC}}$. Finally, it sums up the plaintexts and encrypts the summation using its public key ${\;\mathrm{PK}}_{\mathrm{CC}}$ into $E_{r_{j}}^{2p}$ to form the regional requirement. Therefore, the transmitted message packet from ${\mathrm{BC}}_{j\;}$ to ${\mathrm{NGW}}_{k\;}$ can be represented as ${\mathrm{msg}}_{j}=<{\mathrm{ID}}_{j},\mathrm{Len},E_{r_{j}}^{2p},E_{u_{j}}^{2H}>$. ${\mathrm{BC}}_{j\;}$ reserves the individual power requirement ciphertext $<E_{r_{1}}^{1p}∥E_{r_{2}}^{1p}∥\ldots ∥E_{r_{m}}^{1p}>$ in its database to perform individual power distribution for the next time slot (see Figure 2 for details).

### 5.4. Authentication and Communication in BC, NGW, and CC

CC pre-sends the parameter s as the shared key for the BC, NGW and CC during the initiation stage.

- ${\mathrm{BC}}_{j}$

${\mathrm{BC}}_{j\;}$ computes the HMAC signature ${\mathrm{HMAC}}_{s}\left(j∥k∥t_{j}∥{\mathrm{msg}}_{j}\right)$ using the system master secret s as the key on j, k, and $t_{j}$ and encrypts the message with the public key ${\mathrm{PK}}_{{\mathrm{NGW}}_{k\;}}$. Then it transmits the message to the corresponding ${\mathrm{NGW}}_{k}$.

- ${\mathrm{NGW}}_{k}$

The ${\mathrm{NGW}}_{k\;}$, upon receiving ${\mathrm{ENC}}_{{\mathrm{PK}}_{{\mathrm{NGW}}_{k\;}}}(j∥k∥t_{j}∥{\mathrm{HMAC}}_{s}\left(j∥k∥t_{j}∥{\mathrm{msg}}_{j}\right)$, first verifies the freshness of $t_{j}$ and then re-computes ${\mathrm{HMAC}}_{s}\left(j∥k∥t_{j}∥{\mathrm{msg}}_{j}\right))$. When the decrypted message equals the received one, it decrypts ${\mathrm{ENC}}_{{\mathrm{PK}}_{{\mathrm{NGW}}_{k}}}$ with ${\mathrm{SK}}_{{\mathrm{NGW}}_{k\;}}$ to obtain ${\mathrm{msg}}_{j}$. After obtaining ${\mathrm{msg}}_{j}$, ${\mathrm{NGW}}_{k}$ aggregates all $E_{u}^{2H}$ of its child nodes into $E_{u}^{3H}$ and concatenates $E_{r}^{2p}$ for all the BC nodes to generates ${\mathrm{msg}}_{k}=<{\mathrm{ID}}_{k},\mathrm{Len},E_{r_{1}}^{2p}∥E_{r_{2}}^{2p}∥\ldots ∥E_{r_{n}}^{2p}∥E_{u}^{3H}>$ where $E_{u}^{3H}=\mathrm{Homomorphic}$ addition ($E_{u_{1}}^{2H},\;\ldots ,E_{u_{n}}^{2H}$), and $E_{r_{j}}^{2p}$ denotes the total regional power requirement for ${\mathrm{BC}}_{j\;}$. Then, it computes the HMAC signature ${\mathrm{HMAC}}_{s}\left(k∥\mathrm{CC}∥t_{k}∥{\mathrm{msg}}_{k}\right)$ using the system master secret s and encrypts it with the public key ${\mathrm{PK}}_{\mathrm{cc}}$. Finally, it transmits the aggregate message to the CC.

- CC

After decryption and verification, the CC obtains ${\mathrm{msg}}_{k}$ from p NGWs and then aggregates the p groups of $E_{u}^{3H}$ into $E_{u}^{H}$ = homomorphic additions ($E_{u_{1}}^{3H},\;\ldots ,E_{u_{p}}^{3H}$) and concatenates the p groups of <$E_{r_{1}}^{2p}∥\ldots ∥E_{r_{n}}^{2p}$>. Therefore, the message received and stored in CC database is denoted as $<E_{r_{1}}^{2p}∥\cdots ∥E_{r_{n}}^{2p}∥\ldots ∥E_{r_{1}}^{2p}∥\cdots ∥E_{r_{n}}^{2p}∥E_{u}^{H}>$, as shown in Figure 4.

### 5.5. Power Distribution Generation

The CC decrypts p groups of <$E_{r_{1}}^{2p}∥\ldots ∥E_{r_{n}}^{2p}$> into p groups of <$S_{1},S_{2},\ldots ,S_{n}$> (where $S_{i}\;\mathrm{is}\;\mathrm{the}\;\mathrm{ith}\;\mathrm{regional}\;\mathrm{station}\;\mathrm{requirement}\;\mathrm{summation}$). Then, the CC combines it with $E_{u}^{H}$ to generate p groups of $<R_{1},R_{2},\ldots ,R_{n}>$ (${\mathrm{where}\;R}_{i}$ is the ith regional power distribution ratio). Next, it encrypts p groups of $<R_{1},R_{2},\ldots ,R_{n}>$ with ${\mathrm{PK}}_{\mathrm{BC}}$ and sends them to the p ${\mathrm{NGWs}}_{\;}$, respectively. The NGW relays the ratios to each BC. ${\mathrm{BC}}_{j}$ decrypts the ratio ciphertext with ${\mathrm{SK}}_{{\mathrm{BC}}_{j}}$ and retrieves the previously stored < $E_{r_{1}}^{1p}$, ${\;E}_{r_{2}}^{1p}$, …, ${\;E}_{r_{m}}^{1p}$> from its database. ${\mathrm{BC}}_{j}$ decrypts these values and computes m users’ power distribution $<D_{1},D_{2},\ldots ,D_{m}>$ ($D_{i}=r_{i\;}\cdot R_{j}$) (where $r_{i\;}$ is the individual requirement plaintext) and encrypts them into $\;<E_{1},E_{2},\ldots ,E_{m}>$ (where $E_{i}$ is the ciphertext of ${\;D}_{i}$ with ${\mathrm{SK}}_{{\;\mathrm{HSM}}_{i}})$. Then, it transmits them to every HSM.${\;\mathrm{HSM}}_{i}$ decrypts the power distribution message using its private key and obtains its power distribution for the next time slot.

## 6. Security Analysis

In this section, through a security analysis, we show that the proposed EPPRD achieves all the security goals defined in Section 3.2 and finally we prove EPPRD’s security using the plaintext indistinguishability game.

### 6.1. Mutual Authentication and Data Integrity

In EPPRD, ${\;\mathrm{HSM}}_{i}$ encrypts $g^{a}$ with ${\mathrm{BC}}_{j}\text{’}s$ public key, which ensures that only ${\mathrm{BC}}_{j}$ can recover $g^{a}$ if the employed public key system is secure. Using the same reasoning, $g^{b}$ is only received by real ${\;\mathrm{HSM}}_{i}$ if the public key encryption technique is secure. After ${\;\mathrm{HSM}}_{i}$ receives $g^{a},\;{\mathrm{BC}}_{j}$ is authenticated by ${\;\mathrm{HSM}}_{i}$ because only the real ${\mathrm{BC}}_{j}$ can send $g^{a}$ to ${\;\mathrm{HSM}}_{i}$. Thus, the scheme provides mutual authentication among GWs and the CC.

The randomly generated shared key $K_{\mathrm{ij}}$ ensures the data authentication and integrity between HSM and BC, because an external or internal attacker (of an HSM or NGW) has no authority to access other node’s databases to transmit invalid data. In [21], if the pre-sent shared key s is compromised by an attacker, that attacker may be authenticated by BC with s and launch a man-in-the-middle attack. In contrast, in our scheme, even if the shared key $K_{\mathrm{ij}}$ is compromised, the attacker still cannot be authenticated by the BC or NGW and the secrecy of previous keys remains intact because our authentication scheme provides perfect forward secrecy.

### 6.2. Protection against Eavesdropping Attack

The confidentiality of our scheme is based on the RSA and Paillier encryption algorithms. During authentication, $g^{a}$ and $g^{b}$ are encrypted with RSA encryption between ${\mathrm{HSM}}_{i}$ and ${\mathrm{BC}}_{j}$. In upward transmission, the power consumption message is aggregated using Paillier encryption, and the requirement message is concatenated and encrypted with RSA encryption ${\mathrm{PK}}_{\mathrm{CC}}$.

An attacker located in a HAN can eavesdrop on the communication flow between HSM and BC. However, even if the attacker eavesdrops on the ciphertext $E_{r_{i}}^{1p}$ from ${\mathrm{HSM}}_{i}$ to BC, he cannot recover the individual requirement from ${\mathrm{HSM}}_{i}$ without the private key of ${\mathrm{BC}}_{j}$, and the encrypted individual consumption $E_{u_{i}}^{1H}$ cannot be decrypted without the private key of the CC, because the Paillier encryption’s semantic security resists chosen plaintext attacks.

Similarly, even if an attacker eavesdrops on the communication flow between ${\mathrm{BC}}_{j}$ and the NGW, he cannot obtain the regional requirement and consumption sum other than the individual data, because the regional requirement and consumption sums ($E_{r_{j}}^{2p}$ and $E_{u_{j}}^{2H}$) can only be decrypted using the private key of the CC.

### 6.3. Protection against Internal Attack

There are two possible avenues for internal attacks in the semi-honest model in EPPRD. One is the communication flow between a HSM and a BC and the other is the communication flow between a BC, NGW, and the CC. In the first, messages are intentionally eavesdropped and stored by curious internal participants such as the NGW or another HSM. However, they cannot obtain the individual measurements because they lack the private keys of the BC and CC. The second communication flow may be intentionally eavesdropped on and stored by curious internal participants such as an HSM. However, using this approach, the attacker can only obtain the regional requirement sum and aggregated consumption. Even if he were to have access to the private key of CC, he would not be able to decipher the individual requirement and consumption values.

Therefore, the proposed scheme provides not only confidentiality but also integrity.

### 6.4. Protection against Replay and Man-in-the-Middle Attack

Not only the ciphertext ${\mathrm{ENC}}_{{\mathrm{BC}}_{j}}\left(i∥j∥t_{i}∥g^{a}\right)$ during authentication but also the ciphertext ${\mathrm{ENC}}_{K_{\mathrm{ij}}}(i∥j∥t_{i}∥{\mathrm{HMAC}}_{K_{\mathrm{ij}}}\left(i∥j∥{\mathrm{msg}}_{i}\right)$ in each transmission all contain freshly generated time stamps. Therefore, parties to the communication first verify the freshness of the time stamp and then verify that it is the same time stamp present in the encrypted message. In this way, EPPRD can resist replay attacks.

Consider the communication flow between ${\;\mathrm{HSM}}_{i}$ and ${\mathrm{BC}}_{j}$. After receiving the $g^{a}$ sent by the ${\mathrm{BC}}_{j}$, the ${\;\mathrm{HSM}}_{i}$ can authenticate the ${\mathrm{BC}}_{j}.$ Even if an attacker were to impersonate the ${\mathrm{BC}}_{j}$ or ${\;\mathrm{HSM}}_{i}$, he cannot be authenticated because of the RSA encryption and HMAC signature. Therefore, EPPRD can resist a man-in-the-middle attack.

### 6.5. Security Proof

Since the BC is highly trusted, the security notion of EPPRD focuses mainly on the semi-honest aggregator NGW and HSM. In what follows, we further analyze whether the collusion of the NGW and the compromised HSMs affects the leakage of other users’ privacy, especially requirement and consumption plaintext. The security of EPPRD is based on the cryptosystem and security notion of Paillier.

**Theorem** **1.** Assume semi-honest adversary ADV corrupts the aggregator NGW and at most n − 1 nodes (n is the total number of HSM in a local region), then ADV cannot infer any privacy of other uncompromised users. EPPRD achieves security.

To demonstrate that EPPRD can maintain the plaintext of requirement and consumption, we use the plaintext indistinguishability game described below.

- Setup: The challenger initializes the smart meters set to participant aggregation process. The challenger generates their keys including public and private keys during the secret key generation phase in Section 5.1 and gives the public keys to the adversary.
- Queries: ADV can make “compromise” queries for private keys or plaintext to users. It can compromise at most n − 1 meters. The challenger returns the private key and plaintext of compromised smart meters. ADV may also compromise the aggregator NSM and receives the aggregation from the challenger.
- Challenge: The ADV specifies an uncompromised set U⊆{1,2,…,n}, in which ADV specifies randomly two smart meters ${ℳ}_{0}$ and ${ℳ}_{1}$. The challenger flips a random coin b. If b = 0, the challenger return to the ADV $\left\{E_{r_{j}}^{2p},E_{u_{j}}^{2H}\right\}$, else return {$E_{r_{j}}^{2p\prime },E_{u_{j}}^{2H\prime }$}.
- Guess: The ADV guesses b′∈{0,1}. The ADV wins if b′=b. The advantage of ADV in attacking the scheme is defined as follows:
  (2) $${\mathrm{ADV}}_{ADV}=\left|\mathrm{Pr}\left[b=b\prime \right]-\frac{1}{2}\right|.$$

${\mathrm{ADV}}_{ADV}$ denotes the indistinguishability advantage of ADV. In what follows, we prove the advantage is zero.

**Proof:**  Let us assume the n − 1 nodes are all compromised except for ${\mathrm{HSM}}_{i}$; if the extreme case satisfies the security then it also holds for other cases. We prove that ADV cannot infer the requirement and consumption plaintext of ${\mathrm{HSM}}_{i}$, even if ADV compromises the aggregator NGW and n − 1 HSMs.

The ADV can compromise the NGW and n − 1 HSMs in th query phase and the challenger gives access to the measurement of compromised users or aggregated measurement in NGW as described in Section 5.2:

(3) $$E_{r_{j}}^{2p}=E^{2p}\left(r_{1}+\ldots +r_{i}+\ldots +r_{n}\right)$$

(4) $$E_{r_{j}}^{2H}=E^{2H}\left(u_{1}+\ldots +u_{i}+\ldots +u_{n}\right).$$

In Equation (3), $r_{i}$ refers to the requirement plaintext of ${\mathrm{HSM}}_{i}$ and $u_{i}$ refers to the consumption plaintext of ${\mathrm{HSM}}_{i}$ in Equation (4). Assume the ${\mathrm{HSM}}_{i}$ is the only smart meter that is not compromised by  ADV, so the other nodes’ requirement and consumption do not contribute to the security; Equation (3) can also be written as

(5) $$E_{r_{j}}^{2p}=E^{2p}\left(r_{i}+\sum_{j\neq i}r_{j}\right).$$

Equation (5) is encrypted with ${\mathrm{PK}}_{\mathrm{CC}}$, and the ADV does not know ${\mathrm{SK}}_{\mathrm{cc}}$, so it cannot learn about $r_{i}$. Similarly, Equation (4) can be written as

(6) $${\;E}_{u_{j}}^{2H}=E^{2H}\left(u_{i}+\sum_{j\neq i}u_{j}\right)=E^{2H}\left(u_{i}\right)+E^{2H}\left(\sum_{j\neq i}u_{j}\right).$$

Equation (4) can be written as Equation (6) according to the addition homomorphic property of Paillier; however, the ADV still cannot learn about $u_{i}$ even if $E^{2H}\left(u_{i}\right)$ can be inferred because of the cryptographic measurement.

From Equations (5) and (6), we can conclude that the  ADV cannot correctly infer the requirement and consumption plaintext even if it compromises the aggregator NGW and at most n − 1 HSMs. So the security of ${\mathrm{HSM}}_{i}$ can be guaranteed.

## 7. Performance Analysis

A SG communication system has resource constraints and stringent security requirement that make it difficult to perform computation-intensive operations such as symmetric public cryptographic operations. Furthermore, limited communication bandwidth may lead to delays or latency. Therefore, we analyze our scheme in terms of the communication volume, computational overhead, and delay time.

We fix the number of users at 1 million. The number of NGWs is 50, there are 100 BCs, and we vary the number of HSMs per BC from 1 to 200 with a step size of 20 to study the impact of the numbers of HSMs on communication, computational overhead, and memory consumption. To accommodate the highly frequent need for DS communications in SGs, we first adopt a HAN message transmission interval of 10 s, denoted by Δ, for validating the above performance analysis. Furthermore, we investigate the impact of different Δ values on communication. Considering the same cryptography and similar authentication platforms, we compare the following two schemes performance with ours.

- The no-consumption aggregation scheme. In this scheme, the BC receives publicly encrypted consumption messages $E_{u}^{P}$ rather than homomorphic encryption from all the HSMs and transmits them to the CC via NGW. The CC decrypts the encrypted messages based on its public key successively rather than decrypting the message once as in our scheme. As we can imagine, the no-consumption aggregation scheme requires excessive communication overhead, and its security is not rigorous enough because it lacks the protection of homomorphic encryption.
- The no-regional-requirement aggregation scheme in [21]. In this scheme, the homomorphically encrypted power requirements estimating the future time period and commitments are transmitted upward. In these messages, the commitment is the evidence of the user power consumption plan at each billing period. Thus, it obtains the same requirement object for individual users as in our scheme. However, as described in the Related Works (Section 2), we propose some improvements from various perspectives.

### 7.1. Communication Volume

In the hierarchical architecture, we evaluate the communication volume performance from encryption and authentication overheads by considering the handshake step and the traffic payload through every GW during transmission.

We assume the time slot size and the GWs identities occupy 128 bits/16 bytes, while RSA encryption is 1024 bits/128 bytes for a public/private key pair, the size of the Hash MAC is set to 16 bytes based on MD5 and Paillier encryption is 4096 bits/512 bytes. Therefore, the encryption overhead for the consumption and requirement messages of ${\;\mathrm{HSM}}_{i}$ is 512 and 128 bytes, respectively, and can be completed during the preprocessing phase.

Encrypting ${\mathrm{ENC}}_{{\mathrm{pk}}_{\mathrm{BCj}}}\left(i∥j∥t∥g^{a}\right)$ requires 176 bytes and ${\mathrm{ENC}}_{{\mathrm{pk}}_{\mathrm{HSMi}}}\left(i∥j∥t_{j}∥g^{a}∥{\;g}^{b}\right)$ requires 304 bytes. Transmitting $\left(g^{b},i\right)$ requires 144 bytes, and ${\mathrm{ENC}}_{\mathrm{kij}}\left(i∥j∥t_{i}∥{\mathrm{msg}}_{i}∥\mathrm{HMAC}(i∥j∥t_{i}∥{\mathrm{msg}}_{i}\right))$ requires 1392 bytes. Therefore, the total size of transmissions during communication between one ${\;\mathrm{HSM}}_{i}$ and ${\mathrm{BC}}_{j}$ is 2016 bytes in our scheme. In contrast, ${\mathrm{ENC}}_{{\mathrm{PK}}_{\mathrm{BC}}}\left(E_{i}∥H_{i}∥C_{i}∥{\mathrm{HMAC}}_{S}\left(E_{i}∥H_{i}∥C_{i}\right)\right)$ between one ${\mathrm{HSM}}_{i\;}$ and ${\mathrm{BC}}_{j}$ in the scheme in [21] requires 1424 bytes when m = 1 (m is the time period in [21]). Obviously, our communication overhead between ${\mathrm{HSM}}_{i}$ and ${\mathrm{BC}}_{j}$ is larger than that of the scheme in [21], as shown in Figure 3.

**Figure 5:**
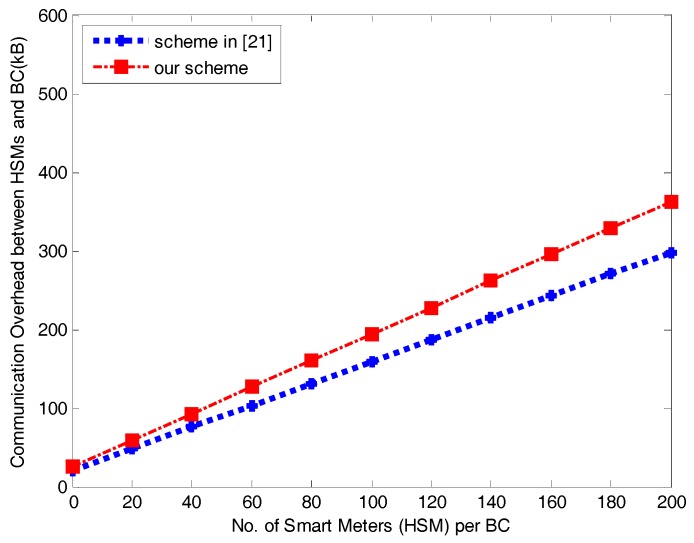
Communication overhead between HSM and BC in the power requirement stage.

Figure 5 plots a comparison of the communication required by our scheme and [21] between any BC and all HSMs. The regional overhead at a BC in our scheme exceeds that of the scheme in [21] slightly due to our more rigorous authentication process during the handshake period and the additional aggregated consumption report.

**Figure 6:**
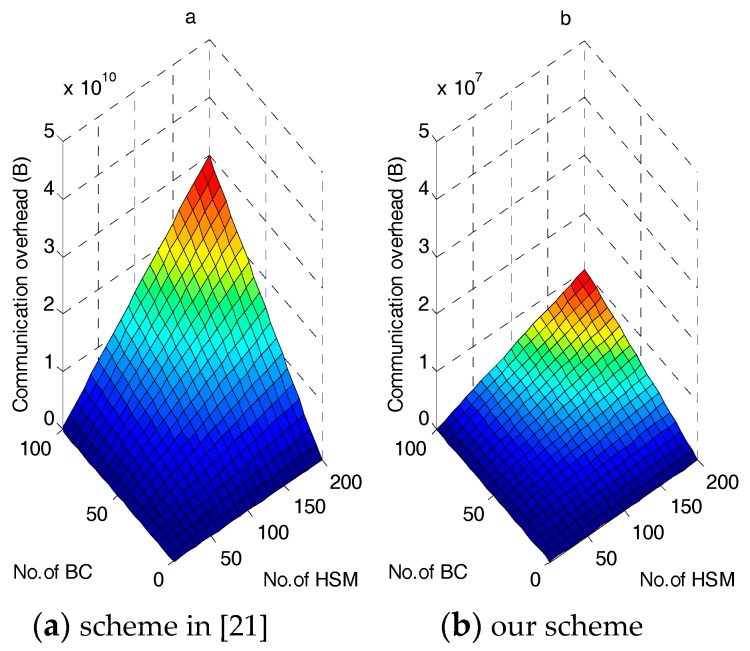
Total communication overhead in the power requirement stage.

However, as shown in Figure 6, this additional overhead has little effect on the overall communications compared with [21]. In fact, Figure 6 shows that our scheme outperforms [21] in terms of overall communications overhead. Figure 6a shows how the communication overhead of [21] changes when the number of HSMs increases. The total system communication overhead increases significantly, and approaches 30 GB when the number of HSM per BC nears 200 and number of BCs nears 100. In contrast, as shown in Figure 6b, the amplitude of growth for our proposed scheme is not large and the total communication never exceeds 11 MB. This result occurs because every transmitted upward message includes a requirement message, an individual commitment packet and a hash packet in [21], but our scheme stores these in the BC and performs an upward transmission of only one regional requirement and one encrypted consumption message. Moreover, our scheme uses symmetric encryption, while the scheme in [21] adopts asymmetric encryption among GWs and the CC, which requires more bytes. The results show that the regional requirement storage/aggregation at the BC and the power consumption aggregation play an important role in reducing the total communication cost and memory consumption.

### 7.2. Computation Overhead

In this evaluation, we ignore the computation overhead involved in the preparation phase because it can be performed offline. The following performance evaluation and analysis combine the authentication and privacy preservation processes.

We performed the experiments based on the FriendlyARM [29] library and the library from [21] using a computer with a processor running at 2.5 GHz, 4 MB of RAM 4 MB and 1 MB of flash memory. The results not only consider message authentication but also privacy preservation issues, although our requirement may be higher than that required for conventional smart meters.

To consume the 160 MH of the BC, we expanded the experimental values by 16 times, including the encryption and decryption time. We adopted the Paillier cryptosystem with 512 bits of modulus and at least $1-2^{-64}$ certainty of prime generation for homomorphic encryption and decryption [28] and for RSA we used a 1024-bit key for asymmetric encryption, decryption [30]. For AES we used a 128-bit key for symmetric encryption and decryption and the MAC is based on the RIPEMD-128 MD5 algorithm, which provides greater resilience against collision and pre-image attacks than does MD5 [31]. The time cost of all primitive operations is listed in Table 2. Based on the test results, we compare the computation cost.

- For ${\;\mathrm{HSM}}_{i}$:

Encrypting $\left(g^{a}\right)$ with ${\;\mathrm{PK}}_{{\mathrm{BC}}_{j}\;}$ for transmission to ${\mathrm{BC}}_{j}$ requires RSA encryption and Diffie-Hellman encryption successively, namely, $2\times T_{\mathrm{aenc}}$, and decrypting encrypted messages from ${\mathrm{BC}}_{j}$ requires one $T_{\mathrm{adec}}$, Computing $K_{\mathrm{ij}}$ and ${\mathrm{HMAC}}_{K_{\mathrm{ij}}}$ requires one $T_{\mathrm{hash}}$ and one $T_{\mathrm{hmac}}$. Therefore, one intact authentication process requires $2\times T_{\mathrm{aenc}}+T_{\mathrm{adec}}+T_{\mathrm{hash}}+T_{\mathrm{hmac}}.$ Encrypting $(i∥j∥t_{i}∥{\mathrm{HMAC}}_{K_{\mathrm{ij}}}\left(i∥j∥{\mathrm{msg}}_{i}\right)$ requires one $T_{\mathrm{aenc}}$. In addition to encrypting the consumption and requirement message packet, denoted as $E^{H}$ and $E^{p}$, respectively, requires $T_{\mathrm{henc}}+T_{\mathrm{aenc}}$ which can be done during the preprocessing stage, Therefore, the total time required is $3T_{\mathrm{aenc}}+T_{\mathrm{adec}}+T_{\mathrm{hash}}+T_{\mathrm{hmac}}$.

- For ${\mathrm{BC}}_{j}$:

The authentication process between ${\mathrm{HSM}}_{i}{\;\mathrm{and}\;\mathrm{BC}}_{j}$ costs the ${\mathrm{BC}}_{j}$ $2\times T_{\mathrm{adec}}+T_{\mathrm{aenc}}+T_{\mathrm{hash}}+T_{\mathrm{hmac}}$. Decrypting a message requires one $T_{\mathrm{adec}}$ and decrypting m $E_{r_{i}}^{1p}$ requires one $\left(m-1\right)\times T_{\mathrm{adec}}$ for summation. Encrypting the summation into $E_{r_{j}}^{2p}$ requires one $T_{\mathrm{senc}}$, and aggregating all the $E_{u_{i}}^{1H}$ messages into $E_{u_{j}}^{2H}$ takes ($m-1)\times T_{\mathrm{mul}}$. Then, ${\mathrm{BC}}_{j}$ takes one $T_{\mathrm{hmac}}$ to generate the HMAC signature and one $T_{\mathrm{senc}}$ to encrypt $\left(j∥k∥t_{j}∥{\mathrm{HMAC}}_{s}\left(j∥k∥t_{j}∥{\mathrm{msg}}_{j}\right)\right)$ with shared key s. Therefore, the total time is $T_{\mathrm{aenc}}\;+T_{\mathrm{senc}}+\left(m+2\right)T_{\mathrm{adec}}+T_{\mathrm{hash}}+2T_{\mathrm{hmac}}+\left(m-1\right)T_{\mathrm{mul}}$.

- For ${\mathrm{NGW}}_{k}$:

Re-computing the HMAC signature ${\mathrm{HMAC}}_{s}\left(j∥k∥t_{j}∥{\mathrm{msg}}_{j}\right)$ requires one $T_{\mathrm{hmac}}$, Decrypting ${\mathrm{ENC}}_{s}\left(j∥k∥t_{j}∥{\mathrm{HMAC}}_{s}\left(j∥k∥t_{j}∥{\mathrm{msg}}_{j}\right)\right)$ requires one $T_{\mathrm{sdec}}$. Upon receiving an $E_{u_{j}}^{2H}$ and aggregating it into $E_{u_{k}}^{3H}$ takes $\left(n-1\right)\times T_{\mathrm{mul}}$. Then, forming ${\mathrm{HMAC}}_{s}\left(k∥\mathrm{CC}∥{\mathrm{msg}}_{k}\right)$ takes $T_{\mathrm{hmac}}$, and encrypting it with ${\mathrm{PK}}_{s}$ for the CC takes one $T_{\mathrm{senc}}$. Therefore, the total time is $T_{\mathrm{senc}}+{\;T}_{\mathrm{sdec}}+2T_{\mathrm{hmac}\;}+\left(n-1\right)T_{\mathrm{mul}}$.

- For the CC:

Upon receiving ${\mathrm{ENC}}_{s}(K∥\mathrm{CC}∥t_{K}∥{\mathrm{HMAC}}_{S}\left(k∥\mathrm{CC}∥{\mathrm{msg}}_{k}\right)$, re-computing the HMAC signature takes one $T_{\mathrm{hmac}\;}$ and decrypting it takes one $T_{\mathrm{sdec}\;}$. Then, aggregating p groups of $E_{u_{k}}^{3H}$ takes $p\times T_{\mathrm{mul}}$, and it takes one ${\;T}_{\mathrm{hdec}\;}$ to receive the total aggregation. Therefore, the total time is ${\;T}_{\mathrm{sdec}\;}+T_{\mathrm{hmac}\;}+p\times T_{\mathrm{mul}}+T_{\mathrm{hdec}\;}$.

**Figure 7:**
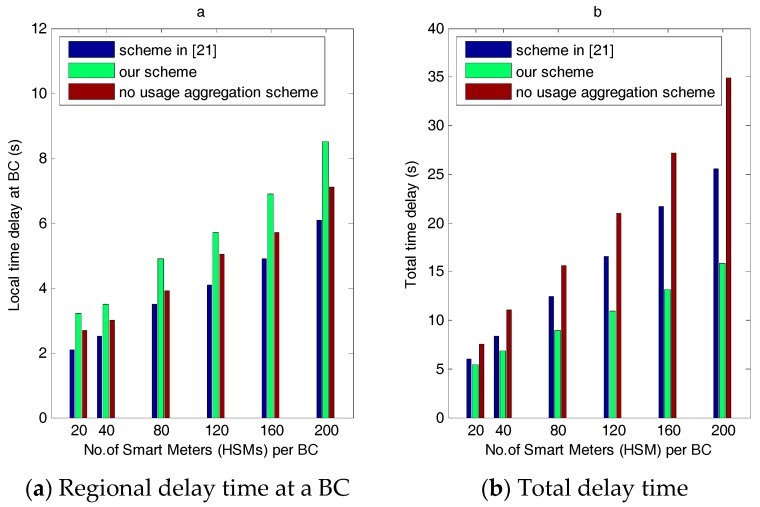
The computation time delay for our scheme, the scheme in [21] and the no-consumption aggregation scheme in the power requirement stage.

According to the above time analysis, combined with the other two schemes, Figure 7 shows the communication time delay of the three schemes in the power requirement stage. Figure 7a shows the change of regional time delay for the three schemes as the number of HSMs increases. When the number of users is 20, the employed method in [21] costs 2.17 s, the no-usage aggregation scheme costs 2.78 s, and our scheme costs 3.18 s. The increasing amplification of the three schemes is 12.1%, 13.4%, and 18.65%, respectively. As we can see, the computation overhead of our scheme is always higher slightly than the other two, and its amplification increases slightly because bidirectional authentication costs more time during the handshake period than the other two schemes. Moreover, the other two schemes do not require regional decryption at the BC for each individual requirement. The no-consumption aggregation scheme adopts the same authentication process as ours, but it does not require the decryption process; therefore, its communication time delay is less than ours. The time delay of the scheme in [21] is smallest because it uses only one session key throughout the authentication process and does not require a bidirectional session key generation process between an HSM and a BC, nor does it require the decryption process at the BC. Therefore, [21] has the least communication time delay cost at a BC of the three schemes.

However, as shown in Figure 7b, regional delay time has little effect on the overall time delay of our scheme compared with the other two schemes. On the contrary, it shows that our scheme outperforms the other two schemes in terms of the overall time delay overhead. Figure 7b shows a comparison of the total delay time. As shown, the total delay increases as the number of users increases; however, the amplification is obviously different. When the number of users is 20, [21] costs 6.2 s, the no usage aggregation scheme costs 8.4 s, and our scheme costs 5.1 s. However, when the number of users is 200, the delay time of the other two increases significantly: the delay time for no-usage aggregation scheme approaches 34.8 and that of [21] is 25.1, while our scheme costs only 16.2 s, which indicates that the effect of regional time delay is insignificant compared to the time delay during the overall communications between the BC, NGW, and CC. It is easy to conclude that the time delay in the latter communication occurs mainly from decryption. In the no-usage aggregation, the individual usage data is not decrypted at the BC; instead, it is transmitted upward to the CC via NGW; consequently, the CC must decrypt all the individual usage data, which costs much time. The scheme in [21] does not aggregate regional requirement data; therefore, it needs to be decrypted by the CC, which is costlier than our scheme. Assume that m, n, and p stand for the number of HSMs per BC, BCs per NGW, and NGWs per CC, respectively. Then, the decryption time complexity degree is o(2 m·n·p) in the no-consumption aggregation scheme, o(m·n·p) in [21], and ours is o(m·n + n·p) during communication between the BCs, NGWs, and the CC. Moreover, from Figure 7b, we can conclude that the regional decryption and aggregation approach involves less total time delay compared to the decryption amounts required in the other two schemes.

Therefore, we can conclude that regional requirement storage and homomorphic aggregation play important roles in reducing the total communication and computation overhead.

### 7.3. Memory Occupancy Rate for Different Transmission Intervals ∆

**Figure 8:**
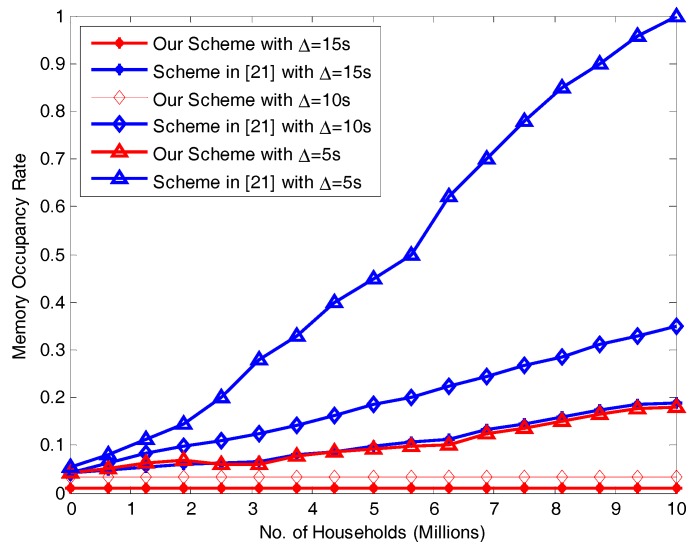
Comparison of memory use between our scheme and [21] with different ∆ values.

The memory required by our scheme and the scheme in [21] with different numbers of users at varying transmission intervals is shown in Figure 8. When ∆ is 15 s and 10 s, our scheme’s memory usage is relatively small. It increases slightly (but no more than 0.16) as the number of users increases. When ∆ is 15 s, the scheme in [21] requires relatively little memory, and it is similar to our scheme when ∆ is 5 s but has an obviously rising trend: eventually, its memory requirement become overwhelming and use up all the available memory. This result demonstrates our scheme’s good performance. This is due to the fact that BCs share lots of processing queue and aggregate fewer processing queue at CC.

### 7.4. Affected Householders with Different Numbers of Attackers

**Figure 9:**
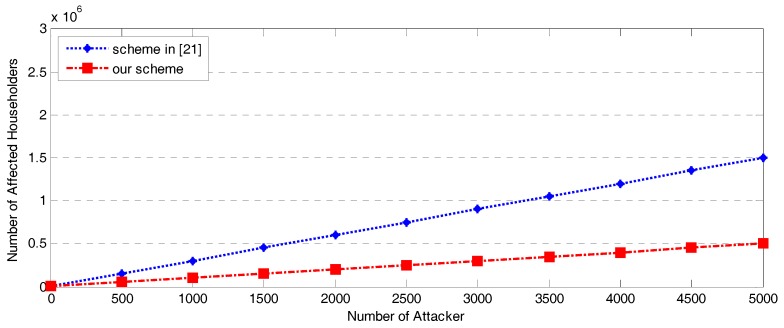
Affected householders in our scheme and the scheme in [21] with different numbers of attackers.

Finally, we show the strictness of our bidirectional authentication by performing attacks in an SG network. We assume that householders are affected if the message they transmit upward is not the same as the one received by the BC, NGW, and CC. We evaluate our authentication by varying the number of SG attackers. We assume the number of households can be up to 3 million, while the number of attackers reaches 5000 at most. We also introduce man-in-the-middle attacks into the SG network and study the number of affected householders with a randomly generated authentication key and a fixed authentication key at the BCs. We distribute 10 attackers into 10 different BCs. As shown in Figure 9, the number of affected householders continues to increase as the number of attackers increases in both the scheme from [21] and our scheme; however, the number of affected householders in our scheme is always lower than the number affected in [21], which does not use a randomly generated authentication key. This result demonstrates that using a randomly generated authentication key would strengthen the privacy preservation of the scheme [21] and help prevent man-in-the-middle attacks. It also shows that our scheme reduces the impact of man-in-the-middle attacks.

## 8. Conclusions

In this paper, we proposed an efficient privacy-preserving power requirement and distribution aggregation scheme for Smart Grid (EPPRD). It is a novelty individual power requirement and distribution scheme while preserving user privacy with a light bidirectional authentication and encryption technique. The existing schemes mostly focus on the total preserving authentication technique or do not consider the whole communication and computation overhead. We locate BC as a regional aggregation station in BAN to aggregate and transmit regional power total and store individual requirement. On the other hand, power consumption in the last time slot is the power distribution reference in the next time slot; its homomorphic encryption scheme together with the authentication scheme ensures the rigorous privacy protection and data integrity. Experiments demonstrate that it plays an important role in reducing computation and communication overhead. In future work, we will further explore low-cost cryptographic algorithms against various attacks and study light cryptographic and authentication algorithms in case there is no trusted model for distributed communication network.

## Figures and Tables

**Table 1:** Notations used in EPPRD.

Symbol	Meaning
CC	Control Center
GW	All Gateways
NGW	Neighborhood Smart Meter
BC	BAN Concentrator
HSM	Home Smart Meter
${\mathrm{PK}}_{\mathrm{CC}}$	Public key of the control center
${\mathrm{SK}}_{\mathrm{CC}}$	Private key of the control center
${\mathrm{HSM}}_{i}$	The ith HSM
${\mathrm{BC}}_{j}$	The jth BC
${\mathrm{NGW}}_{k}$	The kth NGW
${\mathrm{PK}}_{{\mathrm{HSM}}_{i}}$	Public key of ${\mathrm{HSM}}_{i}$
${\mathrm{SK}}_{{\mathrm{HSM}}_{i}}$	Private key of ${\mathrm{HSM}}_{i}$
${\mathrm{PK}}_{{\mathrm{BC}}_{j}}$	Public key of ${\mathrm{BC}}_{j}$
${\mathrm{SK}}_{{\mathrm{BC}}_{j}}$	Private key of ${\mathrm{BC}}_{j}$
${\mathrm{PK}}_{{\mathrm{NGW}}_{k}}$	Public key of ${\mathrm{NGW}}_{K}$
${\mathrm{SK}}_{{\mathrm{NGW}}_{k}}$	Private key of ${\mathrm{NGW}}_{k}$
$E_{r}^{p}$	Public encryption of the requirement for next time slot
$E_{u}^{H}$	Homomorphic encryption of a user’s power consumption
${\mathrm{ENC}}_{\mathrm{key}}\left(M\right)$	Encryption of plaintext M using key
${\mathrm{HMAC}}_{x}\left(M\right)$	HMAC of message M using key x

**Table 2:** Experiment measured average time for each function.

Notations	Descriptions	Time Cost
$T_{a}$	addition	≈0.004 ms
$T_{\mathrm{mul}}$	multiplication	≈0.13 ms
$T_{\mathrm{aenc}}$	asymmetric encryption	≈3.57 ms
$T_{\mathrm{adec}}$	asymmetric decryption	≈0.0032 ms
$T_{\mathrm{senc}}$	symmetric encryption	≈0.0054 ms
$T_{\mathrm{sdec}}$	symmetric decryption	≈0.0014 ms
$T_{\mathrm{henc}}$	Homomorphic encryption	≈2.7 ms
$T_{\mathrm{hdec}}$	Homomorphic decryption	≈0.59 ms
$T_{\mathrm{hash}}$	Hash	≈0.0025 ms
$T_{\mathrm{HMAC}}$	HMAC	≈0.0043 ms

## Abbreviations

The following abbreviations are used in this manuscript:

HSM	User Smart Meter
BC	Building Gateway Smart Meter
NGW	Neighborhood Gateway Smart Meter
GWs	the collection of HSM, BC, and NGW
